# Diseases Admitted as in and Out-Patients under the Care of the Physicians of the Westminster Hospital, from the 20th of August to the 20th of September

**Published:** 1799-10

**Authors:** 


*Difeafes admitted as In and Out-Patients under the care of the Phyficians of the Weftminfter Hofpital, from the 20th of Auguft to the 20th of September.*

| | | | |
|---|---|---|---|
| Fevers - - - | 11 | Gaftrodynia - - | 2 |
| Scarlatina - - - | 1 | Hooping Cough - - | 2 |
| Amenorrhœa - - | 4 | Hemoptoe - - | 4 |
| Anafarca - - - | 5 | Hypochondriafis - | 1 |
| Afcites - - - | 2 | Impetigo - - | 1 |
| Afthenia - - - | 1 | Itch - - - - | 8 |
| Afthma - - - | 3 | Jaundice - - | 1 |
| Catarrh - - - | 1 | Phthifis - - - | 2 |
| Cholera - - - | 1 | Prolapfus - - - | 1 |
| Colic - - - | 1 | Paralyfis - - - | 1 |
| Cough - - - | 3 | Pleurify - - - | 1 |
| Cephalæa - - - | 4 | Quinzey - - - | 1 |
| Diarrhœa - - | 5 | Rheumatifm - - | 7 |
| Dyfpepfia - - - | 2 | Struma - - | 3 |
| Dyfentery - - - | 2 | Urticaria - - - | 1 |
| Dyfuria - - | 1 | Worms - - - | 5 |
| Enterodynia - - | 3 | Vomiting - - - | 3 |

The fevers of this month have diftinctly affumed the bilious character.

---

*** *The third Lift of Difeafes will, if it be found, appear in the next Number; as it has either been miflaid in the Printing-Office, or mifcarried by the Penny-poft.*

